# Effects of short‐term sex steroid suppression on dietary fat storage patterns in healthy males

**DOI:** 10.14814/phy2.13533

**Published:** 2018-01-22

**Authors:** Corey A. Rynders, Stacy L. Schmidt, Audrey Bergouignan, Tracy J. Horton, Daniel H. Bessesen

**Affiliations:** ^1^ Division of Geriatric Medicine University of Colorado Anschutz Medical Campus Aurora Colorado; ^2^ Metropolitan State University of Denver Denver Colorado; ^3^ Division of Endocrinology, Metabolism, and Diabetes University of Colorado Anschutz Medical Campus Aurora Colorado; ^4^ IPHC‐DEPE Université de Strasbourg Strasbourg France; ^5^ UMR 7178 Centre National de la Recherche Scientifique (CNRS) Strasbourg France; ^6^ Department of Art and Art History Colorado State University Fort Collins Colorado; ^7^ Department of Medicine Denver Health Medical Center Denver Colorado

**Keywords:** Energy metabolism, hypogonadism, lipid metabolism, postprandial metabolism, testosterone

## Abstract

Hypogonadism in males is associated with increased body fat and altered postprandial metabolism, but mechanisms remain poorly understood. Using a cross‐over study design, we investigated the effects of short‐term sex hormone suppression with or without testosterone add‐back on postprandial metabolism and the fate of dietary fat. Eleven healthy males (age: 29 ± 4.5 year; BMI: 26.3 ± 2.1 kg/m^2^) completed two 7‐day study phases during which hormone levels were altered pharmacologically to produce a low sex hormone condition (gonadotropin releasing hormone antagonist, aromatase inhibitor, and placebo gel) or a testosterone add‐back condition (testosterone gel). Following 7 days of therapy, subjects were administered an inpatient test meal containing 50 μCi of [1‐^14^C] oleic acid. Plasma samples were collected hourly for 5 h to assess postprandial responses. Energy metabolism (indirect calorimetry) and dietary fat oxidation (^14^CO_2_ in breath) were assessed at 1, 3, 5, 13.5, and 24 h following the test meal. Abdominal and femoral adipose biopsies were taken 24 h after the test meal to determine uptake of the labeled lipid. Postprandial glucose, insulin, free‐fatty acid, and triglyceride responses were not different between conditions (*P* > 0.05). Whole‐body energy metabolism was also not different between conditions at any time point (*P* > 0.05). Dietary fat oxidation trended lower (*P* = 0.12) and the relative uptake of ^14^C labeled lipid into femoral adipose tissue was greater (*P* = 0.03) in the low hormone condition. Short‐term hormone suppression did not affect energy expenditure or postprandial metabolism, but contributed to greater relative storage of dietary fat in the femoral depot. ClinicalTrials.gov Identifier: NCT03289559.

## Introduction

Aging in men is associated with a gradual decline in serum testosterone (T) concentrations at a rate of approximately 0.8% per year after age 30 (Morley et al. 1997; Harman et al. 2001; Feldman et al. 2002). A number of physiological alterations occur concurrently with the age‐related declines in T including the loss of muscle mass, increase in adiposity, and redistribution of body fat to the abdominal visceral depot (Finkelstein et al. 2013; Santosa and Jensen 2014, 2015). Testosterone appears to play a mechanistic role in determining regional body fat distribution in men because most studies (Marin 1995; Katznelson et al. 1996; Snyder et al. 1999; Wittert et al. 2003; Kapoor et al. 2006; Frederiksen et al. 2012) have shown that testosterone supplementation partially prevents the age‐related shifts in body composition. Also, experimental lowering of testosterone concentrations below baseline in eugonadal men increases subcutaneous and deep adipose tissue stores in the thigh and abdomen (Woodhouse et al. 2004). Conversely, experimental elevations in testosterone concentrations above baseline in eugonadal men induces a greater loss of adipose from inter‐muscular stores of the thigh (Woodhouse et al. 2004).

Testosterone likely influences regional adiposity in part through its effects on meal fatty acid (FA) metabolism (Santosa et al. 2008; Host et al. 2013; Santosa and Jensen 2014). However, it is still unclear how modulation of serum T affects the trafficking of dietary FA to oxidation versus storage into upper and lower fat depots. Santosa and Jensen (2012) found that chronically hypogonadal men stored more meal fat (mg per g lipid) in the femoral region compared to eugonadal men. In middle‐aged men who were given 250 mg supplemental testosterone for 5 days, dietary fat uptake in the abdominal depot did not change, but decreased in omental and retroperitoneal adipose tissue (Marin et al. 1996), and in hormonally deficient men, 2 years of testosterone supplementation increased meal fat uptake into upper body subcutaneous adipose tissue (Koutsari et al. 2009). The variability in the results from these few studies may be due to the inconsistency in dose of testosterone given, length of supplementation, initial hormonal condition of the patients, as well as timing of the biopsies taken following ingestion of labeled dietary fat.

To address the limitations of previous studies, we used a model of acute sex steroid suppression‐repletion (GnRH antagonist + placebo or testosterone replacement) to determine the independent effects of serum T concentrations on dietary FA oxidation and storage in upper versus lower fat depots. Because circulating T can be converted to estrogen (E_2_) by aromatization, we also administered an aromatase inhibitor (AI) to suppress E_2_ synthesis thereby minimizing any effects that the presence of estrogen may have on dietary fat metabolism, and creating greater consistency of testosterone levels within and between subjects. Determining the effects of T (and E_2_) on meal FA metabolism could help explain sex‐based differences in regional fat distribution and how alterations in sex hormone status (e.g., andropause, menopause, aging) may lead to an unfavorable distribution of fat. Our hypothesis was that lower T would result in decreased total and dietary fat oxidation and greater meal fat uptake in the lower body (thigh) depot.

## Materials and Methods

### Subjects and preliminary assessments

Eleven nonobese healthy men (BMI 23–29.9 kg/m^2^) aged 25–45 years and not highly trained (<5 h of moderate intensity, planned exercise per week) completed this randomized placebo controlled cross‐over study. Subjects were recruited between 2009 and 2013 for participation by responding to advertisements from the University of Colorado Anschutz Medical Campus (Table 1). The study was approved by the Colorado Multiple Institutional Review Board (COMIRB) and informed written consent was obtained from all participants prior to enrolling in the study. All clinical investigations were conducted according to the principles expressed in the Declaration of Helsinki.

**Table 1:** Subject characteristics

Variable	
N	11
Age (year)	29.4 ± 4.6
Height (m)	1.8 ± 0.1
Body mass (kg)	81.3 ± 11.6
BMI (kg/m^2^)	26.1 ± 2.1
Fat‐free mass (kg)	61.7 ± 9.4
Fat mass (kg)	19.1 ± 5.0
Body fat percentage (%)	23.4 ± 5.0
Trunk fat (kg)	9.0 ± 0.3

Preliminary assessments were completed at the Clinical and Translational Research Center (CTRC) at the University of Colorado Anschutz Medical Campus. At baseline, all subjects underwent a physical examination and biochemical testing to exclude medical illness. Specific exclusions included past or present history of cardiovascular disease, high blood pressure, diabetes, hormonal imbalance and/or metabolic abnormality, medications known to affect weight or lipid metabolism, and body weight change of 5% or greater in the past 6 months. Body composition was measured at baseline using dual energy X‐ray absorptiometry (Hologic Delphi‐W instrument, Waltham, MA).

### Outpatient Protocol

Following preliminary assessments, subjects completed a randomized cross‐over study consisting of two 7‐Day phases. On Day 1 of each phase, sex steroids were altered by the administration of a GnRH antagonist (Cetrotide®, 3 mg initial, 0.25 mg/day thereafter), an aromatase inhibitor (Arimidex, 1 mg/day), and either a transdermal testosterone supplement (testosterone gel 5.0 g containing 50 mg of T/day) or an identical placebo gel. These medication combinations resulted in either a low hormone condition (GnRH_ANT _+ AI + P) or a T replete condition (GnRH_ANT _+ AI + T), completed in a randomized order. The subjects were blinded to which phase of the study was being conducted (i.e., the placebo gel looked similar to the testosterone gel). Also, the study principal investigator was blinded to whether the subject was completing the low testosterone or testosterone replacement phase. The randomization scheme was generated and maintained by the University pharmacy. The average wash‐out duration between conditions was 65 days (minimum = 42 day; maximum = 112 day).

On Days 2 and 5, subjects visited the CTRC to have their blood drawn so that hormone concentrations could be measured 10–12 h after they took their medications. On Days 4–7, subjects consumed a control diet provided by the CTRC metabolic kitchen. The diet composition was 30% fat, 15% protein, and 55% carbohydrate. Energy intake on the controlled diet days was designed to maintain energy balance and was calculated by estimating resting metabolic rate from fat‐free mass (determined via a DXA scan) multiplied by an activity factor as follows: [(23.9 × FFM in kg) + 372] × 1.6.

### Inpatient protocol

**Figure 1:**
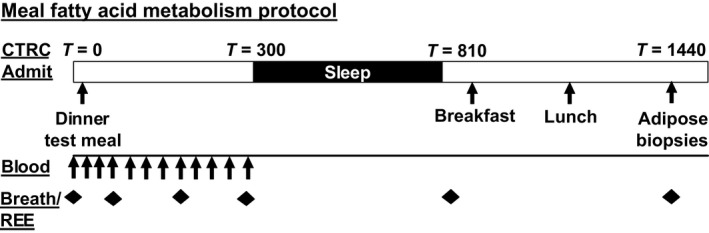
Study design.

Subjects were admitted to the University Hospital CTRC in the evening of Day 6 for both study conditions (Fig. 1). A liquid test meal was consumed at ~5 pm consisting of 35% of daily energy needs (30% fat, 15% protein, 55% carbohydrate). The total fat, carbohydrate, and protein content of the test meal was approximately 32.9 ± 3.9 g, 142.2 ± 17.3 g, and 38.8 ± 4.7 g, respectively. In addition to the liquid meal, approximately 50 *μ*Ci [1‐^14^C] oleic acid (Moravek Biochemicals, Inc., Brea, CA) in olive oil was added to a slice of bread. Blood sampling for insulin, glucose, free fatty acids (FFA), and triglycerides (TG) were collected prior to (*T* = 0) and following the test meal at *T* = 20, 40, 60, 90, 120 150, 180, 210, 240, 270, and 300 min. Indirect calorimetry + ^14^CO_2_ breath samples were collected prior to the test meal and at *T* = 60, 180, 300, 810, and 1440 min following the test meal. To collect breath samples, subjects exhaled through a tube, connected to a 1‐way filter, into a scintillation vial containing 1 mL of 1 mol/L benzathonium hydroxide and 2 mL of absolute methanol, with phenolphthalein (10 mg/100 mL) as an indicator. Scintillation fluid (ScintiSafe™ Plus 50% Cocktail, Fisher Chemical, Waltham, MA) was added to the collection vials, and radioactivity in breath was measured via scintillation counting with a Beckman LS6500 counter (Horton et al. 2002).

Subcutaneous abdominal and femoral adipose biopsies were taken 1440 min (24 h) after the test meal using a “mini‐liposuction” procedure (Bastard et al. 1994). Prior to consuming the test meal in the second study phase (either GnRH_ANT _+ AI + P or GnRH_ANT _+ AI + T, whichever study phase came second), baseline (*T* = 0) adipose biopsies were obtained to determine background ^14^C that may have been retained from the previous test meal. For the femoral region biopsy, the subcutaneous fat found on the upper lateral thigh was taken, and for the abdominal region, the biopsy was taken from the subcutaneous fat in the peri‐umbilical region. The collected adipose tissue samples were frozen immediately in liquid nitrogen and stored at −80°C.

### Assays and calculations

Dietary fat oxidation was determined by multiplying the ^14^CO_2_ specific activity (disintegrations per minute per millimole) by the CO_2_ production rate measured at each time point by indirect calorimetry (millimole per minute) (Bergouignan et al. 2014). The specific activity of the test meal (μCi of ^14^C/g of fat) was used to convert this value to grams of dietary fat oxidized. Adipose tissue lipids were extracted from the tissue samples using standard procedures (Folch et al. 1957), and weighed to the nearest 0.1 mg. The ^14^C content of these lipid samples was then measured with a Beckman LS6500 scintillation counter (Horton et al. 2002) and specific activity was calculated (dpm/g of lipid) for samples from each adipose depot. For samples taken during the second study condition, the ^14^C content obtained at *T* = 0 was subtracted from the activity measured at 24 h. Adipose tissue specific activity (disintegrations per minute per gram) was divided by the meal specific activity (disintegrations per minute per milligram) to determine the relative uptake of tracer into the abdominal and femoral fat depots (disintegrations per minute per gram adipose lipid).

### Statistical analysis

Separate linear mixed effects models with treatment, treatment sequence, and period as fixed factors and subject as a random factor were used to test for differences between the low hormone and T replete conditions. The primary analyses aimed to determine whether differences exist in meal FA uptake between abdominal and femoral depots in low hormone and testosterone replete states. Metabolic (glucose, insulin, FFA, and triglyceride), indirect calorimetery, and meal fatty acid oxidation time course data were reduced to single area under the curve (AUC) values and were included in secondary analyses. Data are presented as mean ± SEM unless otherwise indicated. Differences were defined as statistically significant at *P* < 0.05. Data were analyzed using the PROC MIXED procedures of SAS version 9.0 (SAS Inst. Inc., Cary, NC).

## Results

### Subjects

Subject characteristics are displayed in Table 1. Eleven nonobese, young males with normal testosterone levels completed the randomized cross‐over study. One subject opted out of the fat biopsy procedures, and the study team was unable to obtain a femoral fat biopsy on another subject, thus meal fat storage data are presented for *n* = 10 (abdominal meal fat uptake) and *n* = 9 (femoral fat uptake). There were no adverse events, harm to subjects, or unintended effects of the hormone treatments. The most common feedback from the subjects was that that they felt tired and had less energy, but these symptoms were not reported more frequently in the placebo group as compared to the T replacement conditions.

### GnRH_ANT _+ AI + P versus GnRH_ANT _+ AI + T

Following the GnRH_ANT _+ AI + P intervention, serum T (−7.7 ± 1.8 nmol/L, *P* < 0.001) and E_2_ (−57.6 ± 13.6 pmol/L, *P* < 0.001) were decreased compared to baseline (Table 2). Testosterone concentrations in the add‐back condition (GnRH_ANT _+ AI + T) were not significantly different from baseline (−0.5 ± 1.3 nmol/L, *P* > 0.05), while E_2_ levels remained suppressed (−60.9 ± 17.6 pmol/L, *P* < 0.001; Table 2).

**Table 2:** Sex steroid values at baseline and following the interventions

Variable	Sex steroids (Baseline)	Sex steroids (GnRH_ANT _+ AI + P)	Sex steroids (GnRH_ANT _+ AI + T)
Testosterone (nmol/L)	18.2 ± 4.6	10.2 ± 5.1^a^	17.9 ± 4.2
Testosterone (%reduced/%restored)	**–**	−41.1 ± 28.6	+100.6 ± 29.7
Estradiol (pmol/L)	110.1 ± 46.4	51.1 ± 22.1^a^	48.0 ± 19.6^a^
Estradiol (%reduced)	**–**	−47.3 ± 23.0	−45.3 ± 36.4
T:E_2_ ratio	0.21 ± 0.13	0.20 ± 0.08	0.43 ± 0.17^a^

Values are means ± SD.

a Represents significant differences compared to baseline (<0.05)

### Postprandial metabolic and hormonal responses

**Figure 2:**
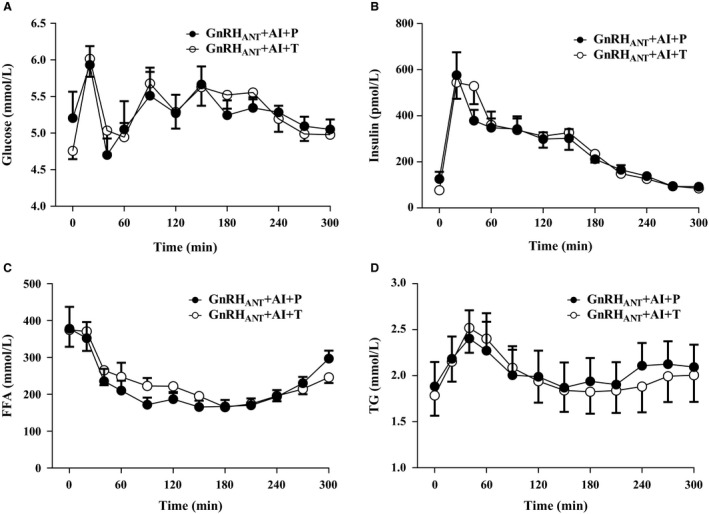
Glucose (A), insulin (B), FFA (C), and TG (D) responses measured over 5 h (300 min) following administration of the test meal at *t* = 0 min under conditions of sex hormone suppression (GnRH_ANT__ _+ AI + P) and testosterone add‐back (GnRH_ANT__ _+ AI + T). Values are mean ± SEM.

The 5 h time course of glucose (*P* = 0.76), insulin (*P* = 0.48), FFA (*P* = 0.22), and TG (*P* = 0.74) were comparable between the GnRH_ANT _+ AI + P and GnRH_ANT _+ AI + T conditions (Fig. 2). We separately examined the area under the curve for the first 2 h and last 3 h of post‐test meal responses and these segments were also not different between conditions (*P* > 0.05, Fig. 2).

### Energy expenditure and fat oxidation

**Figure 3:**
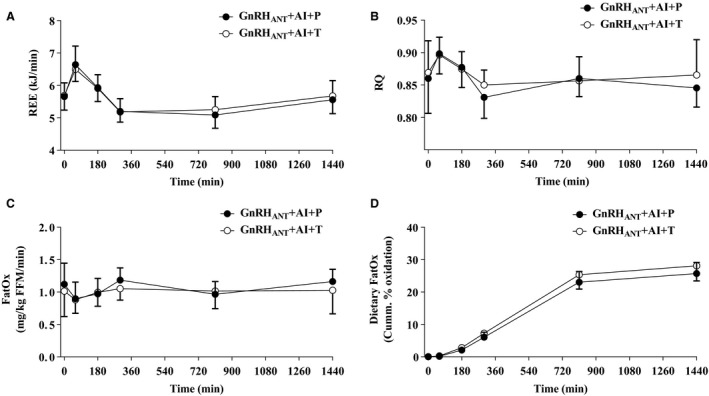
Resting energy expenditure (A), respiratory quotient (B), whole body fat oxidation (C), and cumulative dietary fat oxidation (D) responses measured for 24 h following administration of a test meal at *t* = 0 min under conditions of sex hormone suppression (GnRH_ANT__ _+ AI + P) and testosterone add‐back (GnRH_ANT__ _+ AI + T). Values are means ± SEM. REE, resting energy expenditure; RQ, respiratory exchange ratio, FatOx, whole body fat oxidation.

Resting energy expenditure (REE), respiratory quotient (RQ), and whole‐body fat oxidation as measured by indirect calorimetry were not different between conditions over the initial 5 h following the test meal (Fig. 3A–C; REE, *P* = 0.49; RQ, *P* = 0.86; Fat oxidation, *P* = 0.71) or when measured the following morning (*t* = 810 min post‐test meal; REE, *P* = 0.23; RQ, *P* = 0.70; Fat oxidation, *P* = 0.84) and 24 h after the test‐meal (REE, *P* = 0.34; RQ, *P* = 0.23; Fat oxidation, *P* = 0.14). The cumulative oxidation of meal fatty acids (fraction of meal fat) over 24 h was not different between the GnRH_ANT _+ AI + P (25.9 ± 1.6%) and GnRH_ANT _+ AI + T (28.2 ± 2.3%) conditions (*P* = 0.12, Fig. 3D).

## 24 h meal fat storage

**Figure 4:**
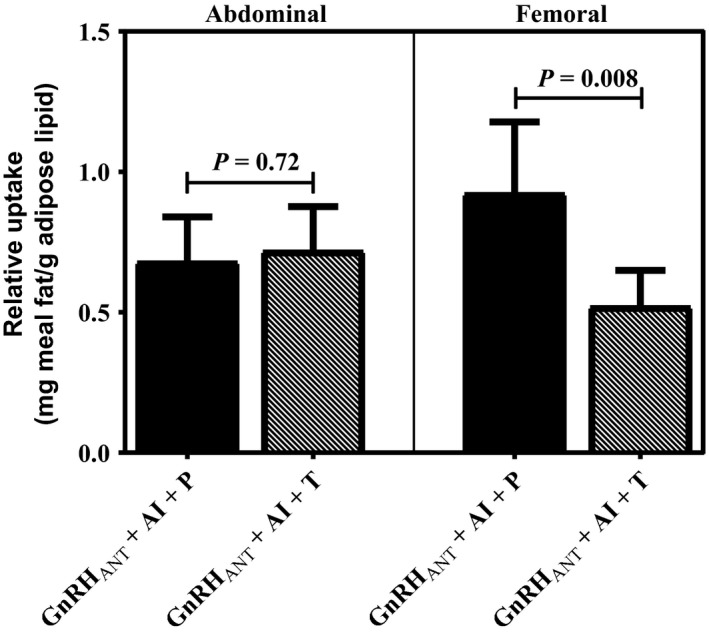
Relative 24‐h dietary fat uptake into abdominal and femoral lipid depots following short‐term sex hormone suppression (GnRH_ANT__ _+ AI + P) and testosterone add‐back (GnRH_ANT__ _+ AI + T). Values are means ± SEM.

Average meal fatty acid storage (mg meal FA/g adipose lipid) in the abdominal depot was not significantly different between conditions (*P* = 0.72, Fig. 4). However, the proportion of meal FA stored in femoral adipose tissue was significantly greater in GnRH_ANT _+ AI + P than GnRH_ANT _+ AI + T (*P*= 0.008, Fig. 4). We also assessed the ratio of the adipose lipid specific activity in abdominal to femoral fat. The ratio of meal FA storage in abdominal to femoral fat was less in the GnRH_ANT _+ AI + P condition than the GnRH_ANT _+ AI + T condition (1.04 ± 0.15 vs. 1.65 ± 0.23, *P* = 0.03) indicating that in the low hormone condition there was preferential storage of dietary fat in the lower body fat region compared with the testosterone add‐back condition.

## Discussion

We studied the effects of short‐term sex hormone suppression plus placebo or testosterone add‐back on postprandial metabolism, energy expenditure, dietary fat oxidation and storage into abdominal versus femoral fat depots. Our results demonstrated that 7 days of low T (compared to T replete condition) had no impact on postprandial metabolic responses to a mixed meal, energy expenditure, or whole body substrate oxidation. However, short‐term sex steroid suppression did result in trends for reduced dietary fat oxidation and significantly increased uptake dietary fat uptake into the femoral depot.

### Postprandial metabolism

Low testosterone in men is an independent risk factor for development of metabolic syndrome, which is characterized by dyslipidemia, insulin resistance, and abdominal (visceral) obesity (Rao et al. 2013). It is unclear whether the dysmetabolism observed in the hypogonadal state is due to T deficiency, or age‐related changes in adiposity. To isolate the effects of T from the confounding effects of aging, we used a model of short‐term sex steroid suppression in combination with placebo or testosterone add‐back and performed measurements of basal and postprandial metabolism. Using this experimental approach, we found that concentrations of FFA, TG, insulin, and glucose were similar in the basal state and showed similar responses to a 5‐h mixed meal challenge in low T compared to T replete conditions.

Our results corroborate and extend the findings of a randomized, double‐blind, placebo‐controlled, crossover study of T on circulating metabolites and hormones by Host et al. (2013). In this study, 12 healthy, young males received gonadotropin‐releasing hormone agonist treatment for 1 month with placebo or T add‐back for 1 day to produce high or low physiological concentrations of T. During the 1‐day placebo or T add‐back, subjects participated in a 5‐h basal period and a 3 h hyperinsulinemic‐euglycemic clamp. The authors reported that concentrations of TG, VLDL‐TG, FFA, insulin, glucose, and glucose infusion rates were comparable during both basal and insulin stimulated periods among the various T manipulations. Another study by Santosa and Jensen (2012) comparing 12 men with chronic hypogonadism and 13 control men matched for age and body composition showed similar meal insulin responses and meal fatty acid absorption/transport into the circulation between eugonadal and low‐T groups. Taken together, the insulin resistance and postprandial dysmetabolism commonly observed in hypogonadal men may be driven in a large part by the resultant effects of low T on body composition and fatness.

### Whole body substrate utilization and energy expenditure

Reduced fat oxidation and REE may be early manifestations of male hypogonadism, (Santosa et al. 2010; Host et al. 2013) which would lead to increased TG uptake and storage and the gradual development of insulin resistance over time. However, contrary to our original hypothesis, low T did not reduce REE or whole body fat oxidation. These results are in agreement with a study by Santosa et al. (2010) which measured REE and the RQ by indirect calorimetry after a 3 week period of sex hormone suppression/repletion and reported that suppression of testosterone and/or estrogen did not change REE and RQ compared to maintenance of physiological concentration. The study by Santosa et al. (2010) is particularly relevant to this study because it used an aromatase inhibitor (2.5 mg/day of letrozole) to prevent the conversion of T to E_2_ during the period of suppression and hormone add‐back. Others studies that have examined the effects of T on EE and substrate metabolism over similar periods of time have also reported no effect (Braun et al. 2005; Host et al. 2013). Together, the results of our study and previously published studies strongly suggest that the effects of sex steroids on EE and substrate oxidation are most likely mediated by body composition changes although a primary role for the ratio of T/E_2_ cannot be excluded.

### Meal fat oxidation and storage

Body fat distribution is clearly affected by testosterone, but exactly how testosterone channels the preferential storage of fat to different adipose tissue beds has not been clearly defined. Our results and the results of prior studies suggest that one way T may be affecting body fat distribution is through its effects on the trafficking of dietary‐derived fatty acids towards storage (Santosa and Jensen 2014). Specifically, we show that short term sex steroid suppression resulted in a trend for reduced dietary fat oxidation and significantly increased dietary fat uptake into the femoral depot. These experimental data confirm the results of a cross‐section study by the Jensen group showing that hypogonadal men stored a greater proportion of both dietary FA and FFA in lower body subcutaneous fat than did eugonadal men (Santosa and Jensen 2012). Our data also agree with another recent study from the Jensen group demonstrating that 4 weeks of GnRH agonist treatment to lower sex hormones resulted in greater femoral adipose tissue meal fat uptake compared to a group that received GnRH agonist plus testosterone gel add‐back (Santosa et al. 2017). The detailed studies of Jensen's group suggested that the greater femoral fat uptake in the low hormone condition was due to increased lipoprotein lipase (LPL) and acetyl coA synthetase (ACS) activity. Unfortunately, we did not explore protein concentrations or gene expression of relevant markers of adipose tissue storage or uptake in this study.

### Strengths and limitations

A strength of this study is that we used an aromatase inhibitor in combination with the GnRH antagonist to minimize any effects that the presence of estrogen may have on dietary fat metabolism, and creating greater consistency of testosterone levels within and between subjects. Approximately 60% of estradiol in men is derived from the testes and conversion of androgens by aromatization. There is some evidence to support a role for E_2_ in regional fat distribution in males. For example, inactivation of estrogen receptor‐*α* or aromatase genes leads to increased fat mass in male mice (Heine et al. 2000; Jones et al. 2000). In an aged orchidectomized male rat model, 17beta‐estradiol supplementation preserved lean muscle and prevented fat accumulation (Vandenput et al. 2002). Finally, in men with prostate cancer, bicalutamide monotherapy (a drug treatment that increases both estrogen and testosterone) lessens fat accumulation compared to treatment with a gonadotropin‐releasing hormone agonist (Smith et al. 2004). Furthermore investigation is necessary to determine the relative role of E_2_ in regulating body composition in men.

A main limitation in our design was that we did not have a ‘control’ condition where meal FA metabolism was studied without manipulation of sex steroids. Another limitation is that although administration of GnRH antagonist reduced the mean T concentration by 41%, it produced a suppression level only slightly below the threshold for low T (<300 ng/mL). The range of suppression was also wide (−7% to −91%) which might explain why modest effects were observed in whole body and meal fat storage.

## Conclusions

In conclusion, compared to a normal T level, 7 days of low testosterone does not affect oxidation of dietary fat, energy expenditure, or postprandial nutrient metabolism. However, it may contribute to a greater relative storage of dietary fat in femoral fat depots.

## Conflict of Interest

The authors have no conflict of interest to declare.
